# Evaluation of a pragmatic approach to predicting COVID-19-positive hospital bed occupancy

**DOI:** 10.1136/bmjhci-2024-101055

**Published:** 2025-02-05

**Authors:** Derryn Lovett, Thomas Woodcock, Jacques Naude, Julian Redhead, Azeem Majeed, Paul Aylin

**Affiliations:** 1 Department of Primary Care and Public Health, Imperial College London, London, UK; 2 Chelsea & Westminster Hospital NHS Foundation Trust, London, UK; 3 London North West Healthcare NHS Trust, London, UK; 4 University of the Witwatersrand Johannesburg, Johannesburg, South Africa; 5 Imperial College Healthcare NHS Trust, London, UK

**Keywords:** COVID-19, Public Health, Decision Support Systems, Management, Electronic Health Records, Health Services Research

## Abstract

**Study objectives:**

This study evaluates the feasibility and accuracy of a pragmatic approach to predicting hospital bed occupancy for COVID-19-positive patients, using only simple methods accessible to typical health system teams.

**Methods:**

We used an observational forecasting design for the study period 1st June 2021 to –21st January 2022. Evaluation data covered individuals registered with a general practitioner in North West London, through the Whole Systems Integrated Care deidentified dataset. We extracted data on COVID-19-positive tests, vaccination records and admissions to hospitals with confirmed COVID-19 within the study period. We used linear regression models to predict bed occupancy, using lagged, smoothed numbers of COVID-19 cases among unvaccinated individuals in the community as the predictor. We used mean absolute percentage error (MAPE) to assess model accuracy.

**Results:**

Model accuracy varied throughout the study period, with a MAPE of 10.8% from 12 July 2021 to 18 October 2021, rising to 20.0% over the subsequent period to 15 December 2021. After that, model accuracy deteriorated considerably, with MAPE 110.4% from December 2021 to 21 January 2022. Model outputs were used by senior healthcare system leaders to aid the planning, organisation and provision of healthcare services to meet demand for hospital beds.

**Conclusions:**

The model produced useful predictions of COVID-19-positive bed occupancy prior to the emergence of the Omicron variant, but accuracy deteriorated after this. In practice, the model offers a pragmatic approach to predicting bed occupancy within a pandemic wave. However, this approach requires continual monitoring of errors to ensure that the periods of poor performance are identified quickly.

**WHAT IS ALREADY KNOWN ON THIS TOPIC**

- Many published studies have applied and reviewed state-of-the-art modelling techniques for predicting bed occupancy during the COVID-19 pandemic. However, very few studies set out to evaluate pragmatic models designed with typical health system analysis teams in mind.

**WHAT THIS STUDY ADDS**

- This study established that it is feasible to deploy a pragmatic model predicting bed occupancy for COVID-19-positive patients at a regional level, with a reasonable degree of accuracy. Furthermore, the resulting predictions were useful in planning and operational delivery of care during the pandemic.

**HOW THIS STUDY MIGHT AFFECT RESEARCH, PRACTICE OR POLICY**

- The model evaluated in this study could be deployed in future pandemics where more sophisticated models are not feasible. More broadly, the findings of this study support collaboration between research teams and healthcare providers in developing, implementing and evaluating modelling practices on a regional and local level, as well as the importance of using and reporting model error metrics when sharing, discussing and publishing results.

## Background

 During the COVID-19 pandemic, effective hospital bed management was essential because of the increased demand for acute care among patients with severe infections. Demand for beds was driven by large numbers of infected patients, which necessitated additional healthcare protection measures to keep staff and patients safe.^1^,^3^ Health systems faced increasing demand for the beds, staff and equipment needed to manage patients effectively, requiring additional ‘surge’ capacity. This temporary additional capacity took the form of repurposed ward space within the existing hospital estates and new sites opening to care for patients with COVID-19.^4 5^ Given that such additional capacity is expensive and takes time to open, the accurate predictions of demand can be valuable in planning and managing resources effectively.^6 7^

Previous studies on predicting bed occupancy due to COVID-19 fall into two broad categories. First, those using an epidemiological modelling approach based on the susceptible–exposed–infected–recovered (SEIR) model or its variants, focusing on infections severe enough to lead to hospitalisation.^8^,^12^ Second, those combining a model predicting the number of admissions with a model predicting length of stay for admitted patients to yield a predicted bed occupancy.^13^,^18^ One study used linear regression to predict future bed occupancy based on the current and past hospital bed occupancy.^19^ While some of these studies demonstrated good predictive accuracy, the statistical knowledge and experience required to apply and interpret these models are often absent in local health systems.^20^

In North West London (NWL), the National Health Service (NHS) response to the pandemic was coordinated and strategically led by the COVID-19 NWL Gold Command group, where senior representatives from across the integrated care system would come together at least once a week to look at operational and strategic actions, which were required to support the system. This study evaluates models developed to support this group in decision-making, in particular, to focus resources where needed during the pandemic. These models, initially developed within an NHS analyst team, were predicated on the idea that COVID-19 vaccines are protective against hospital admission for COVID-19, and that, therefore, hospital bed occupancy for COVID-19 is influenced by the number of unvaccinated individuals in the population.^21^,^23^ Furthermore, older people are known to be more susceptible to severe COVID-19, and hence more likely to be admitted to hospital.^24 25^ This study aimed to develop and evaluate the effectiveness of pragmatic predictive models for COVID-19-positive bed occupancy in hospital trusts, using information on the number of reported COVID-19 cases in the community and the proportion of the population vaccinated.

## Methods

### Study design

We used an observational forecasting design for the study period 1 June 2021–21 January 2022. This period coincided with two large waves of COVID-19 infection in the UK, driven by the Delta SARS-CoV-2 variant in the summer of 2021 and the Omicron variant in the winter of 2021–2022. The research question we sought to answer was: how accurately can we predict future hospital bed occupancy by fitting a linear regression model on the number of cases in the community some fixed number of days previously? The underlying hypothesis is that if the rate of severe disease in the community, and the distribution of length of stay in hospital, remained sufficiently stable over time, a model could be fit to extrapolate this pattern into the future. We compared a simple model, using only the number of cases of COVID-19 in the community, with a multivariable model incorporating case numbers by age band. Age bands were incorporated to allow for differing hospitalisation rates by age. The first model was run on 12 July 2021, and the final model was run on 11 January 2022.

### Data source

NWL Whole Systems Integrated Care deidentified data hold collated records of 2.2 million residents of NWL^26^ and contain information on COVID-19 community testing, vaccination status and daily hospital bed occupancy from NWL NHS Trust situation reports.^27^ We extracted positive COVID-19 community test results, records of all COVID-19 vaccinations and bed occupancy in NWL hospitals during the study period.

### Participants

We included patients registered with a general practitioner and aged 18 or over at the time the model was run. Inclusion criteria for hospital spells were that the patient occupied a general or acute bed in an NWL hospital at any time during the study period and that the patient was reported as COVID-19-positive in the daily situation report.^28^

### Primary outcome

The primary outcome measure is the mean absolute percentage error (MAPE) in the daily number of COVID-19-positive adults predicted to occupy general and acute hospital beds in NWL hospitals, compared with the number observed.

### Predictor variables

Predictor variables were daily counts of cases of COVID-19 in the community where the individual was not protected by vaccination. Total cases in each age band (18–24, 25–34, 35–44, 45–54 and over 55) were defined as the number of individuals with positive COVID-19 tests with a given sample date. To estimate the number of unprotected cases each day, we took the proportion of the overall population in each age band who were not double vaccinated each day and multiplied this by the total cases in that age band. This yielded an estimate of the cases in people who were not double vaccinated. We added a fixed small proportion of the estimated number of vaccinated cases to allow for imperfect vaccine efficacy. Therefore, at timepoint t in age band *j*, the number of unprotected cases was given by the following formula:

$$\sigma_{j}^{t}\epsilon P_{j}^{t}+\left(1-\sigma_{j}^{t}\right)P_{j}^{t}$$

where σ^*t*^_*j*_ is the proportion vaccinated in age band *j* at time *t*, ϵ is 1 − efficacy of vaccination and *P*^*t*^_*j*_ is the number infected in the population in age band *j* at time *t*. Based on the available evidence, we used a vaccine efficacy of 90% (ϵ=0.1)^29^ (see online supplemental appendix 1 for more details).

We applied a 14-day moving average to smooth these unprotected case numbers to avoid spurious fluctuations impacting the predictions.

### Statistical analysis

Each time the model was run, we split the available data into training and test periods. The training period start date was fixed until model accuracy was reduced due to changes in the relationship between cases in the community and occupied beds. When this occurred, a new start date was set, based on the discussions with clinicians and data patterns, which often conformed with ‘waves’ of the pandemic and vaccination programme. This defined a series of phases each with a fixed starting date for the training period. The end date of the training data advanced as more data became available. Training datasets for phases would overlap, as each phase would use some, but not all the data from the previous phase (see online supplemental appendix 1). The test period was set to be the last 14 days of available data at each point the model was run. We used linear regression models with zero intercept to predict adult general and acute bed occupancy from unprotected cases. The first model was a simple linear regression, using unprotected cases in all age bands as the independent variable. The second model was a multiple linear regression, with one independent variable for each age band. We allowed for a lag between COVID-19 community cases and hospitalisation, by regressing the daily occupancy on unprotected cases between 3 and 10 days prior. This lag is referred to as the prediction horizon. Each time a prediction was required, we fitted models on the training data for each horizon and selected the horizon that yielded the lowest mean square error on the test data. The model with this prediction horizon was then applied to the latest available data to make the predictions for subsequent days. For example, assuming the lowest error was found for 6 days, we then applied this 6-day prediction horizon and made predictions for each of the following 6 days beyond the date of the latest available community case data. We also calculated the 95% prediction interval for each of these daily predictions. New data were made available two times a week a new model was fitted, and the resulting predictions were made available to Gold Command.

### Test of model performance and external validation

Model performance was evaluated in two ways. First, each instance of the model, covering a specific time period, was evaluated once the actual COVID-19-positive bed occupancy counts were available for the dates covered by its predictions. This was done using mean absolute error and MAPE. Second, to evaluate the performance of the overall modelling strategy, the predictions made over the whole study period were compared with the actual occupancy using the same metrics. We also calculated the percentage of predictions within the 95% prediction interval. Where two or more predictions were available for the same date, the prediction from the most recent model was used.

## Results

During the study period, 1 June 2021–21 January 2022, 429 473 COVID-19-positive tests were recorded, with the highest rate of cases in the 18–24-year olds (26 733 per 100 000) and lowest in the over 80-year olds (6085 per 100 000) (table 1). By 21 January 2022, 1 453 108 residents of NWL had received their second dose of vaccine, representing 65.9% of eligible individuals. The percentage of individuals who had received two COVID-19 vaccinations ranged from 86.1% in the oldest age band, offered their first dose on 8 December 2020, to 54.5% in the youngest, offered their first dose on 18 June 2021. This was consistent with national data showing highest vaccine uptake in older people. At the start of the study period in June 2021, the average number of COVID-19 occupied beds in NWL hospitals was below 50, rising to plateau in August with around 200 occupied beds before a rapid increase to 500 COVID-19-positive patients occupying beds by the end of the study.

**Table 1:** COVID-19-positive tests from 1 June 2021 to 21 January 2022 and percentage of NWL population who have received two COVID-19 vaccinations by 21 January 2022

Age band	Count of recorded COVID-19-positive tests(rate per 100 000)	Count of population with two COVID-19 vaccinations(% of age band vaccinated)	Total population
18–24	66 203 (26 733)	134 920 (54.5%)	247 642
25–34	136 643 (24 885)	315 407 (57.4%)	549 091
35–44	97 027 (19 984)	287 535 (59.2%)	485 516
45–54	65 799 (18 416)	251 121 (70.3%)	357 293
55–64	38 633 (14 453)	209 936 (78.5%)	267 292
65–79	20 372 (9318)	186 296 (85.2%)	218 635
80+	4796 (6085)	67 893 (86.1%)	78 818
**TOTAL**	**429 473(19484)**	**1 453 108(65.9%)**	2 204 287

NWLNorth West London

**Figure 1:**
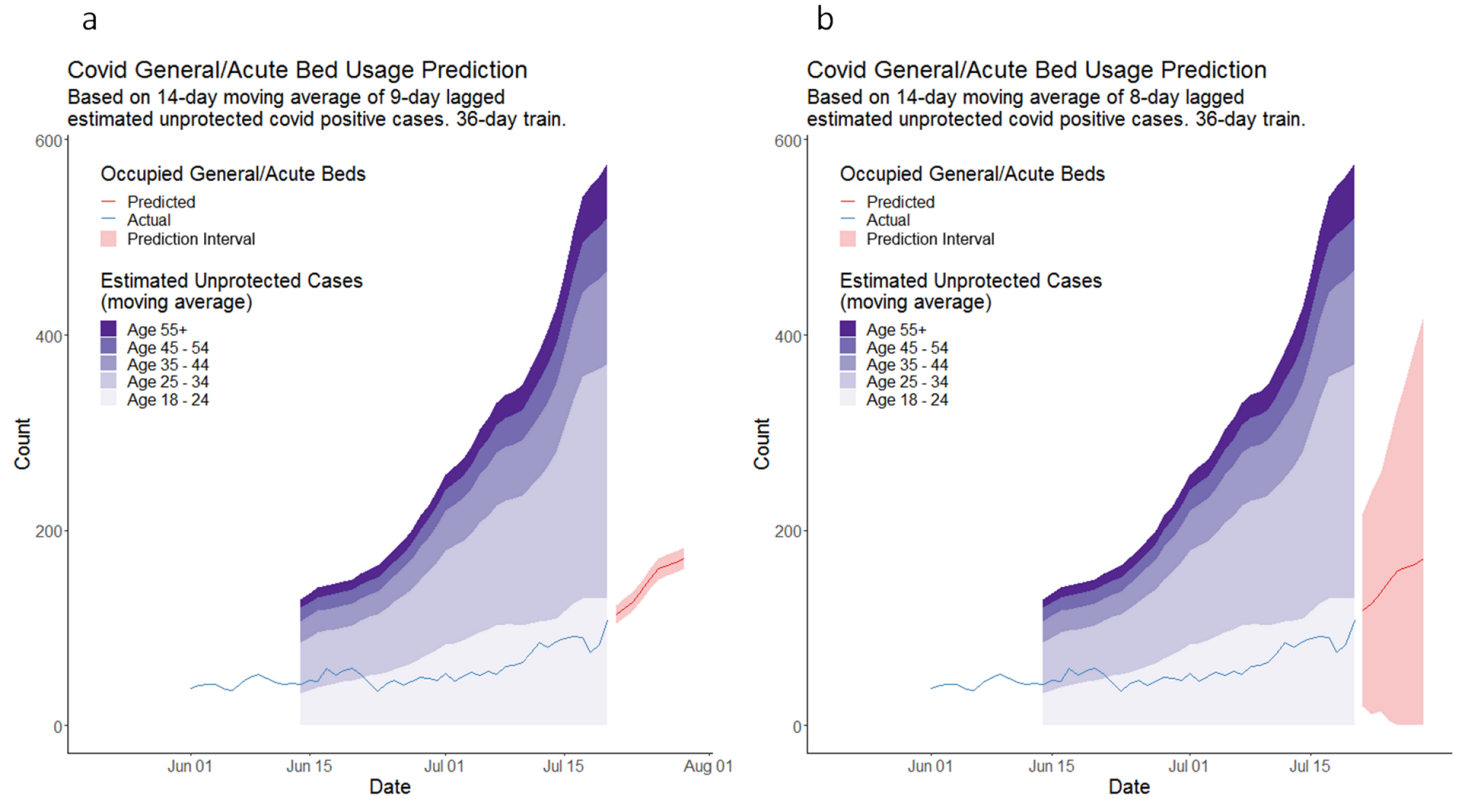
Results of example simple and multivariable models run on 21 July 2021, showing predicted number of COVID-19-positive patients occupying hospital beds in NWL, along with unprotected cases in the community. (**a**) shows results of the simple model and (**b**) the multivariable model incorporating age. The stacked area plots show the estimated number of unprotected cases in the community stratified by age band. The line plots show the actual bed occupancy used to train the model and the subsequent daily predictions made by the model.

Prediction results from simple and multivariable example models are shown in figure 1 and table 2. Both were fitted on 21 July 2021 using all data from the study period before this date. On this occasion, the multivariable model, incorporating age, on average overpredicted by 7.8 occupied beds, with an absolute error of 10.2% of the actual occupancy. The simple model on average overpredicted by 3.3 occupied beds, with an absolute error of 6.3% of the actual occupancy. During the study period, the training period start date for our models changed four times, due to changes in the behaviour of COVID-19 in the population (see online supplemental table 1). Phase 1 ran from 11 December 2020 to 29 April 2021, Phase 2 from 1 June 2021 to 25 October 2021, Phase 3 from 1 August 2021 to 27 February 2022 and Phase 4 from 1 November 2021 to 29 December 2022.

**Table 2:** Example model from 21 July comparison between predicted and observed number of COVID-19-positive hospital beds

Date	Actual	Simple modelNot incorporating age bands			Multivariable modelIncorporating age bands		
		Prediction (95% prediction interval)	Error	Absolute % error	Prediction (95% prediction interval)	Error	Absolute % error
21	113	114 (105 to 123)	1	0.9 %	118 (20 to 217)	5	4.4%
22	110	120 (111 to 130)	10	9.1%	125 (11 to 239)	15	13.6%
23	126	127 (118 to 137)	1	0.8%	136 (14 to 258)	10	7.9%
24	132	138 (128 to 147)	6	4.5%	147 (5 to 290)	15	11.4%
25	136	151 (140 to 161)	15	11.0%	159 (0 to 325)	23	16.9%
26	151	161 (150 to 171)	10	6.6%	162 (0 to 353)	11	7.3%
27	192	164 (154 to 175)	−28	14.6%	165 (0 to 387)	−27	14.1%
28	161	167 (157 to 178)	6	3.7%	171 (0 to 419)	10	6.2%
29	162	171 (161 to 182)	9	5.6%	–	–	–
		**Mean**	3.3	6.3%		7.8	10.2%

### Performance of the overall modelling strategy

**Figure 2:**
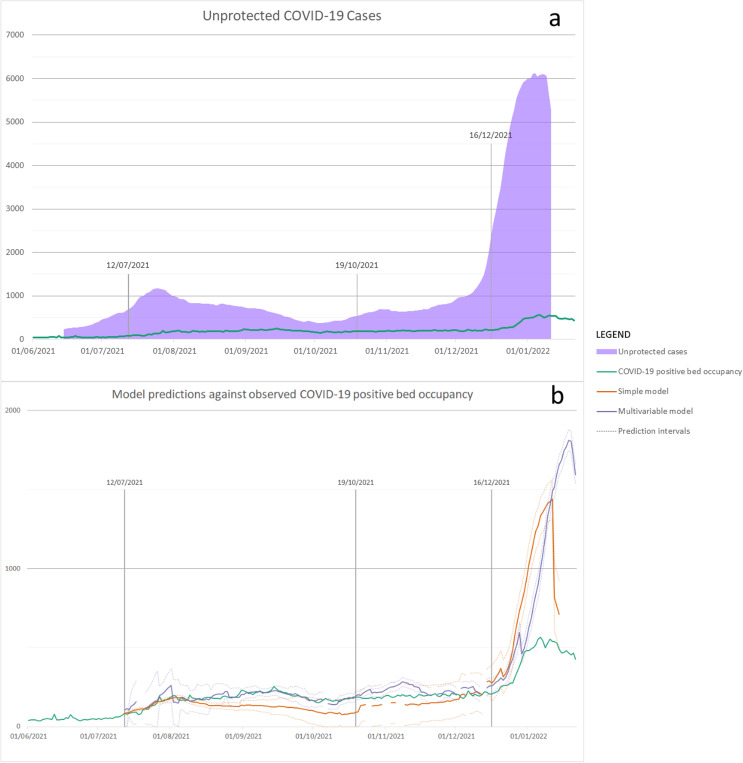
(**a**) Estimated unprotected COVID-19 cases and (**b**) model predictions from simple and multivariable model from 1 June 2021 to 21 January 2022. (**a**) The area plot shows the daily number of estimated unprotected cases in the community, and the line plot shows the actual bed occupancy. Both measures are plotted over the entire study period. (**b**) The solid green line shows the actual general and acute bed occupancy for COVID-19-positive patients across NWL hospitals. The orange and purple lines show the predictions made by the simple and multivariable models, respectively, with solid lines showing the point prediction and dotted lines showing the 95% prediction interval. For each date on the horizontal axis, the prediction plotted is that from the most recently run model before that date. Any breaks in these plotted lines are due to breaks in the model predictive outputs due to changing the model training period or unavailable data.

The first instance of the model was fitted on 12 July 2021 and the last on 11 January 2022. Both predictive models were run on 49 dates during the study period, averaging two times per week. The estimated number of unprotected cases and actual bed occupancy are plotted in figure 2a. The bed occupancy predictions made by each model are shown, along with actual bed occupancy, in figure 2b.

Over all predictions made using the simple model, the MAPE was 45.6% for 12 July 2021–21 January 2022. Similarly, for the multivariable model, the MAPE was 33.3% for this period (table 3). Initially, low error levels increased beyond specific timepoints, with errors remaining low for substantially longer for the multivariable model. Retrospectively, as a part of this study, we used statistical process control analysis to identify the time at which these changes occurred. To do so, we constructed a c-chart and used the standard shift rule of runs of length 8 or more to identify timepoints where errors deviated from the prior stable level.^30^ This identified that 19 October and 16 December were the dates after which there was statistical evidence that the errors for the multivariable model deviated from the previous period. By splitting the results of both models at these timepoints, errors for both models were much lower in the first and second time period compared with the third (table 3). These splits also demonstrated that the multivariable model produced a lower error during the first period, 10.8%, and the second period, 20.0%, with the simple model producing a higher error in both these periods, 29.7% and 22.4%, respectively. The actual COVID-19-positive bed occupancy fell within the multivariable prediction interval on 98% of predictions up to 19 October and a much lower 42%, then 8% in the two following periods.

**Table 3:** Model comparison of predicted number of COVID-19-positive hospital beds over different time periods within the study period

	(a) 12 July 2021–21 January 2022	(b) 12 July 2021–18 October 2021	(c) 19 October 2021–15 December 2021	(d) 16 December 2021–21 January 2022
Model run count	49	26	15	8
Simple model				
MAPE	45.6%	29.7%	22.4%	89.8%
Number of predictions	177	99	48	30
Mean prediction horizon (range)	8.5 (3 to 10)	9.9 (9 to 10)	6.1 (3 to 10)	8.8 (3 to 10)
Mean prediction interval range (coverage*)	149.6 (49%)	77.7 (34%)	234.4 (96%)	248.5 (23%)
Multivariable model				
MAPE	33.3%	10.8%	20.0%	110.4%
Number of predictions	185	95	53	37
Mean prediction horizon (range)	7.5 (3 to 10)	7.5 (3 to 10)	6.8 (3 to 10)	8.8 (3 to 10)
Mean prediction interval range (coverage*)	107.9 (64%)	122.3 (98%)	70.5 (42%)	125.5 (8%)

MAPE rates are shown across the whole study period and split across three subperiods. Since training periods increased as more data were added and were reset when accuracy reduced, these errors are calculated across models fitted using a range of different training periods (online supplemental table 1). Average absolute percentage errors are the percentage errors for each daily prediction against the observed bed occupancy, averaged across the time period. (a) Averages of the whole study period, (b) from 12 July 2021 to 18 October 2021 when error rates increase, (c) from 19 October 2021 to 15 December 2021 and (d) from 16 December 2021 to 21 January 2022. * Mean prediction interval range coverage is the percentage of days on which the observed bed occupancy fell within the prediction interval.

MAPEmean absolute percentage error

All of the statistical analyses, with the exception of this retrospective analysis of the errors, were conducted throughout the study period, as the results were used both to inform the Gold Command group and in monitoring the accuracy of the predictions made (phases 1–4 as described above).

In all instances, the multivariable model produced negative coefficients in some age bands. While it is impossible to calculate variance inflation factors for zero-intercept models, the negative coefficients are likely due to multicollinearity between different age bands experiencing very similar patterns of cases. Experimenting with different age bands did not resolve this issue.

## Discussion

We implemented and evaluated two regression-based approaches to predicting COVID-19 bed occupancy across acute hospitals in NWL during the pandemic’s third (Delta variant) and fourth (Omicron variant) waves. Both models were based on the hypothesis of a stable linear relationship between unvaccinated cases in the community and occupied acute beds several days later. The simple model used total unvaccinated cases, and the multivariable model split these cases across five age bands. The multivariable model outperformed the simple model on MAPE, except during the rapid growth in cases associated with the start of the Omicron wave in London. During that time, neither model performed well. One factor contributing to this may be the different clinical characteristics of the Omicron variant when compared with the Delta variant of the virus.^31^ Another factor is that this model does not account for the protective effect of prior infection against subsequent infection and hospitalisation. This could result in the overestimation of both the number of unprotected individuals and the number of COVID-19-positive patients occupying hospital beds.

The predictions made by the multivariable model for bed occupancy between 12 July and 18 October 2021 had an MAPE of 10.8%, representing a reasonable degree of accuracy for the intended use of managing bed capacity. However, even during the subsequent reasonably stable period, this error doubled. Furthermore, likely multicollinearity in this model may render the coefficients unstable and the model unreliable when applied to subsequent time periods. The lack of an intercept term in the model meant that variance inflation factors were not well defined, making this issue harder to address. This is likely a fundamental limitation of the age-band stratified approach. Predictions made by the simple model were worse on average, with MAPE of 29.7% and 22.4% for the two relatively stable periods. The multivariable model in particular appeared to overpredict bed occupancy after actual occupancy levelled off following a period of increase. This effect was driven by increased numbers of unprotected cases in the community that did not translate into commensurate bed occupancy. This may be due to differences in case severity or virus variants at different stages of each wave and potentially to variations in hospital admission and discharge criteria at high occupancy levels. In principle, such differences could be accounted for by retraining the model to reflect the new system dynamics. However, in practice, this is problematic since, at the point of change, there is insufficient data to capture the new relationship between cases and occupancy.

Previous studies of bed occupancy prediction for COVID-19 use various approaches to model validation and evaluation, making direct comparisons of predictive accuracy difficult. For example, Baas *et al* report a mean absolute error of 1.96 beds for a 5-day prediction horizon at one site and 4.25 at another. Still, they do not report a suitable denominator to convert these to an MAPE.^14^ Ryu *et al* achieved a 3.4% MAPE using a 12-hour prediction horizon.^19^ Bekker *et al* report a weighted MAPE of 8% with a 3-day horizon and 13% with a 7-day horizon,^18^ comparable with our multivariable model’s best performance.

The available data limit the modelling approach described in this study, and now that COVID-19 testing is not universally available free of charge in London, the relationship between reported cases in the community and hospital admissions is unlikely to be stable enough for this approach to work effectively. Even when testing is widely available, as it was at the height of the pandemic, the approach will be sensitive to changes in population behaviour. The model approximates unprotected cases using the overall population coverage of the vaccine for infected cases. However, the bulk of any inaccuracy caused by this approximation is absorbed into the model coefficients, and this approximation is, therefore, unlikely to adversely impact the performance of the model. We used a fixed value from the literature for vaccine efficacy in this evaluation. In practice, this parameter will vary with the type of vaccine given (eg, mRNA (messenger ribonucleic acid) vaccines generally provided better longer term protection against hospitalisation than viral vector vaccines), individual demographic and clinical factors and the dominant virus variant at the time. We did not account for immunity acquired through prior infection with COVID-19; in future work, this protective effect could be incorporated into the model in a similar way to that of vaccination. We did not account for local variations in prevalence within NWL nor bed occupancy at individual hospital sites. Although more geographical granularity might enable increased single-site accuracy, smaller sample size would decrease accuracy as London hospitals do not have fixed catchment areas, with patients exercising choice over where to present.

Despite these limitations, the pragmatic modelling approach evaluated in this study appears to match the accuracy of more involved approaches during the periods of relative stability, although not during periods of more fundamental change. Another strength of the approach is the use only of routinely collected data. This approach may, therefore, be suited to rapid response modelling, where health systems need a ‘good enough’ prediction tool in place quickly and without specialised expertise, perhaps at the beginning of a new epidemic or wave. If used in this way, prediction outputs need to be closely monitored against true bed occupancy to detect when a period of poor predictive accuracy is entered. Rapid increases in MAPE can provide a timely indication of a fundamental change in the epidemiology of the pandemic. Additional parameters could also be agreed between analysts and decision makers, for example, setting a threshold for acceptable error rates. The model can be retrained as needed once a new pattern is established. Where expertise is available for more sophisticated modelling, for example, using modified SEIR-D models, there may be potential to adapt more readily to changes in the nature of the virus or the system response.

All model predictions presented in this study were shared on creation, two times a week, and discussed at weekly GOLD Command meetings, aiding understanding of future pressure on hospital bed occupancy, allowing the safe and coordinated opening and closing of additional COVID-19 prioritised areas to support clinical care across multiple departments.

Our evaluation method closely follows the use of the model in practice, with each model run drawing on the latest available data and repeated runs over an extended period. Many previous studies only evaluate the application of the models in question for one or two model runs or use patient-level cross validation of the data rather than evaluating against actual bed occupancy. Our approach yields a more realistic picture of the likely accuracy of the modelling approach.

Future research should investigate whether allowing the vaccine efficacy parameter to be selected during model fitting would improve this approach. There may be benefits in applying this simple modelling approach to other scenarios, such as predicting pressures on the acute hospital system during winter. Although population-wide testing data are unlikely to be available going forward, a similar approach might be adopted using primary care data to provide an early warning for increased emergency department attendance and hospital admissions. Standardised approaches to evaluating bed occupancy prediction models should also be explored through consensus building methods. The existing literature encompasses disparate methods and metrics, making meaningful comparisons of predictive accuracy difficult.

## Conclusions

Linear regression models may offer a pragmatic rapid approach to predicting hospital bed occupancy based on the community testing data in future waves of the COVID-19 pandemic or other respiratory virus epidemics. While this approach can yield reasonably accurate predictions during relatively stable periods of such an epidemic, it is important to monitor error rates in real time while such a model is in use since predictions can become inaccurate with changes in the virus itself and in population protection against serious illness and hospitalisation, as well as through the societal response, such as the implementation of non-pharmaceutical interventions like home working, social distancing and use of face masks.

## supplementary material

10.1136/bmjhci-2024-101055 online supplemental file 1

## Data Availability

Data may be obtained from a third party and are not publicly available.
