# Supplementary material for: Evaluation of a pragmatic approach to predicting COVID-19-positive hospital bed occupancy
Source: BMJ Health Care Inform. 2025 Feb 5;32(1):e101055. doi: 10.1136/bmjhci-2024-101055 (PMC11800226; doi:10.1136/bmjhci-2024-101055)
Supplement: online supplemental file 1 [file bmjhci-32-1-s001.pdf]

Appendix

Supplementary Appendix 1

This appendix describes in more detail the modelling approach evaluated in this study.

Model Equation

The basic idea of the approach is to relate the bed occupancy Y to an estimate of the number of unprotected cases in the population through a constant of proportionality K:

$$Y = K(\sigma \epsilon P + (1 - \sigma) P)$$

where σ is proportion vaccinated, ϵ is 1 - efficacy of vaccination, and P is the moving average of the number infected in the population. The constant K is the regression coefficient in this simple model, to be fitted to the data. No intercept term is fitted, reflecting an assumption that there would be no beds occupied by COVID-19-postive patients if there are no unprotected cases in the community. Thus allowing for a time lag between testing positive for COVID-19 and admission to hospital, we have:

$$Y_{(t+h)} = K(\sigma_t \epsilon P_t + (1 - \sigma_t) P_t)$$

where t is the timepoint we are predicting from; and h is the prediction horizon - i.e. the difference in time between t and the target time.

To adapt this idea to stratify by age band:

$$\mathrm{Y} = \sum_{\mathrm{j}} \beta_j (\sigma_{\mathrm{j}} \epsilon \mathrm{P}_{\mathrm{j}} + (1 - \sigma_{\mathrm{j}}) \mathrm{P}_{\mathrm{j}})$$

Where Y is the number of beds used, β_j are constants (regression coefficients), σ_j are proportions vaccinated in each age band, ϵ is 1 - efficacy of vaccination. P_j are 14-day moving averages of the number infected in the population in each age band. Again introducing the time lag, this formula becomes:

$$Y^{(t+h)} = \sum_j \beta_j \left(\sigma_j^t \, \epsilon \, P_j^t \; + \; \left(1 \; - \sigma_j^t\right) P_j^t \right)$$

Where t is the timepoint we are predicting from; and h is the prediction horizon - i.e. the difference in time between t and the target time.

Supplementary Table 1

Regression model training periods

Phase	Model Execution Date Range** (Number of model runs)	Training Period	
		Start Date	End Date Range (Number of days)
One	07/07/2021 - 28/10/2021 (30)	11/12/2020*	24/01/2021 - 29/04/2021 (95)
Two	09/11/2021 - 06/01/2022 (18)	01/06/2021*	25/10/2021 - 23/12/2021 (59)
Three	11/01/2022 - 10/03/2022 (13)	01/08/2021†	29/12/2021 - 23/02/2022 (56)
Four	31/03/2022 - 19/04/2022 (4)	01/11/2021†	29/12/2021 - 29/12/2021 (0)

**During each of these date ranges, models were run twice a week except on rare occasions when the data were not updated in time. This was as frequent as possible, since data were never updated more than twice a week during the study. A new phase was triggered when there was evidence of increasing error rates. Each new phase used a new training period comprising only more recent data, as indicated in the training period columns of this table.

*Training period start date remained consistent within the phase.

†Training period start date remained consistent within the phase with two exceptions, once at the end of phase three and once at the beginning of phase four. This adjustment was due to adapting the model to respond appropriately to changing relationships in the model parameters as described in the methods.
